# Baseline C-reactive protein predicts efficacy of the first-line immune checkpoint inhibitors plus chemotherapy in advanced lung squamous cell carcinoma: a retrospective, multicenter study

**DOI:** 10.1186/s12885-023-11737-x

**Published:** 2023-12-16

**Authors:** Xinlong Zheng, Longfeng Zhang, Lin Wu, Jun Zhao, Jianguo Sun, Yong Fang, Jin Zhou, Qian Chu, Yihong Shen, Zhenzhou Yang, Lijin Chen, Meijuan Huang, Xiaoyan Lin, Zhenhua Liu, Peng Shen, Zhijie Wang, Xin Wang, Huijuan Wang, Zhengbo Han, Anwen Liu, Hongmei Zhang, Feng Ye, Wen Gao, Fang Wu, Zhengbo Song, Shengchi Chen, Chenzhi Zhou, Qian Wang, Chunwei Xu, Dingzhi Huang, Xiaobin Zheng, Qian Miao, Kan Jiang, Yiquan Xu, Shiwen Wu, Haibo Wang, Qiuyu Zhang, Shanshan Yang, Yujing Li, Sihui Chen, Gen Lin

**Affiliations:** 1 Department of Thoracic Oncology, Clinical Oncology School of Fujian Medical University, Fujian Cancer Hospital, Fuzhou, China; 2 Fujian Key Laboratory of Advanced Technology for Cancer Screening and Early Diagnosis, Fuzhou, China; 3 The Second Department of Thoracic Oncology, the Affiliated Cancer Hospital of Xiangya School of Medicine, Central South University/Hunan Cancer Hospital, Changsha, China; 4 Department of Thoracic Medical Oncology, Peking University Cancer Hospital and Institute, Beijing, China; 5 Cancer Institute, Xinqiao Hospital, Army Medical University, Chongqing, China; 6 Department of Medical Oncology, Sir Run Run Shaw Hospital, Zhejiang University, Zhejiang, China; 7 School of Medicine, Sichuan Cancer Hospital and Institute, Sichuan Cancer Center, University of Electronic Science and Technology of China, Chengdu, Sichuan China; 8 Department of Oncology, Tongji Hospital, Tongji Medical College, Huazhong University of Science and Technology, Wuhan, China; 9 Department of Respiratory Diseases, The First Affiliated Hospital, College of Medicine, Zhejiang University, Hangzhou, China; 10 Department of Cancer Center, The Second Affiliated Hospital, Chongqing Medical University, Chongqing, China; 11 Department of Oncology, Affiliated Quanzhou First Hospital of Fujian Medical University, Quanzhou, China; 12 Department of Thoracic Oncology, Cancer Center and State Key Laboratory of Biotherapy, West China Hospital, Sichuan University, Chengdu, China; 13 Department of Oncology, Fujian Medical University Union Hospital, Fuzhou, China; 14 Department of Medical Oncology, Provincial Clinical College, Fujian Medical University, Fujian provincial hospital, Fuzhou, China; 15 Department of Oncology, Nanfang Hospital, Southern Medical University, Guangzhou, China; 16 Medical Oncology Department, National Cancer Center/National Clinical Research Center for Cancer/Cancer Hospital, Chinese Academy of Medical Sciences, and Peking Union Medical College, Beijing, China; 17 Department of Oncology, Zhongshan Hospital of Xiamen University, Xiamen, China; 18 Department of Respiratory Medicine, the Affiliated Cancer Hospital of Zhengzhou University, Zhengzhou, China; 19 Department of Oncology, Shengjing Hospital of China Medical University, Shenyang, China; 20 Department of Oncology, The Second Affiliated Hospital of Nanchang University, Nanchang, China; 21 Department of Oncology, Xijing Hospital, Airforce Military Medical University, Xian, Shanxi China; 22 Department of Medical Oncology, Cancer Hospital, The First Affiliated Hospital of Xiamen University, School of Medicine, Xiamen University, Teaching Hospital of Fujian Medical University, Xiamen, China; 23 Department of Medical Oncology, The First Affiliated Hospital with Nanjing Medical University, Nanjing, Jiangsu China; 24 Department of Oncology, The Second Xiangya Hospital, Central South University, Changsha, Hunan China; 25 Department of Clinical Trial, Cancer Hospital of the University of Chinese Academy of Sciences, Hangzhou, Zhejiang China; 26 Department of Oncology, Nanping First Hospital Affiliated to Fujian Medical University, Nanping, China; 27 Respiratory Medicine Department, State Key Laboratory of Respiratory Disease, National Clinical Research Center for Respiratory Disease, Guangzhou Institute of Respiratory Health, The First Affiliated Hospital of Guangzhou Medical University, Guangzhou, China; 28 Department of Respiratory Medicine, Affiliated Hospital of Nanjing University of Chinese Medicine, Nanjing, Jiangsu China; 29 Department of Respiratory Medicine, Affiliated Jinling Hospital, Medical School of Nanjing University Nanjing, Nanjing, Jiangsu China; 30 Department of Thoracic Oncology, Tianjin Medical University Cancer Institute and Hospital, Tianjin, China; 31 Institute of Immunotherapy, Fujian Medical University, Fuzhou, China; 32 Interdisciplinary Institute for Medical Engineering, Fuzhou University, Fuzhou, China

**Keywords:** Lung squamous cell carcinoma, Immune checkpoint inhibitors, C-reactive protein, Predictive biomarker

## Abstract

**Aims:**

To investigate the predictive value of baseline C-reactive protein (CRP) levels on the efficacy of chemotherapy plus immune checkpoint inhibitors (ICI) in patients with advanced lung squamous cell carcinoma (LSCC).

**Materials and methods:**

In this retrospective multicenter study spanning from January 2016 to December 2020, advanced LSCC patients initially treated with chemotherapy or a combination of chemotherapy and ICI were categorized into normal and elevated CRP subgroups. The relationship between CRP levels and treatment outcomes was analyzed using multivariate Cox proportional hazards models and multivariate logistic regression, focusing primarily on the progression-free survival (PFS) endpoint, and secondarily on overall survival (OS) and objective response rate (ORR) endpoints. Survival curves were generated using the Kaplan-Meier method, with the log-rank test used for comparison between groups.

**Results:**

Of the 245 patients evaluated, the 105 who received a combination of chemotherapy and ICI with elevated baseline CRP levels exhibited a significant reduction in PFS (median 6.5 months vs. 11.8 months, HR, 1.78; 95% CI: 1.12–2.81; *p* = 0.013) compared to those with normal CRP levels. Elevated CRP was identified as an independent risk factor for poor PFS through multivariate-adjusted analysis. However, among the 140 patients receiving chemotherapy alone, baseline CRP levels did not significantly influence PFS. Furthermore, within the combination therapy group, there was a notable decrease in the ORR (51% vs. 71%, *p* = 0.035), coupled with a significantly shorter OS (median 20.9 months vs. 31.5 months, HR, 2.24; 95% CI: 1.13–4.44; *p* = 0.033).

**Conclusion:**

In patients with advanced LSCC, elevated baseline CRP levels were identified as an independent predictive factor for the efficacy of combination therapy with chemotherapy and ICI, but not in chemotherapy alone. This suggests that CRP may be a valuable biomarker for guiding treatment strategies.

**Supplementary Information:**

The online version contains supplementary material available at 10.1186/s12885-023-11737-x.

## Introduction

Lung Squamous Cell Carcinoma (LSCC) accounts for approximately 20–30% of all lung cancers, is very difficult to treat, and differs remarkably from lung adenocarcinoma [1]. Driver genes that act as therapeutic targets have primarily been detected in lung adenocarcinoma, while for LSCC, platinum-based chemotherapy has long been promoted as the best first-line treatment [2, 3]. In recent years, several large phase III randomized controlled trials have demonstrated that addition of immune checkpoint inhibitors (ICI) to platinum-based chemotherapy may form a new standard first-line treatment for patients with advanced LSCC [4–7].

Biomarkers that accurately predict response to ICI by metastatic NSCLC are currently lacking, and this is particularly true for LSCC. Biomarkers such as PD-L1 expression and tumoral mutation burden (TMB) can help to select patients who may benefit most from ICI monotherapy [8–11]. However, neither PD-L1 immunohistochemistry staining nor TMB alone is sufficiently accurate to identify potential responders to PD-1/PD-L1 blockade-based immunotherapy in NSCLC [4–6, 8]. Although potential biomarkers have been reported in several studies, they have not been validated through independent cohorts [12, 13].

Inflammation, notably driven by factors such as C-reactive protein (CRP), is thought to participate in cancer immunoresistance by promoting tumor growth and metastasis and activating oncogenic signaling pathways [14, 15]. CRP, synthesized by the hepatocytes in response to proinflammatory cytokines, has been linked to poor prognosis of various cancers including esophageal [16], bladder [17], melanoma [18], and other cancers [19]. However, the role of CRP in LSCC, particularly in the context of ICI plus chemotherapy, is less explored. Previous research has presented inconsistent conclusions about the significance of CRP as a prognostic factor in advanced NSCLC [20, 21]. Some studies have highlighted the predictive value of CRP for ICI monotherapy, but reports remain contradictory [22–26].

From the methodological perspective, it’s crucial to consider whether the biomarker is prognostic or predictive or both. As such, the nature of the association between CRP and clinical benefit in LSCC patients treated with ICI plus chemotherapy requires further investigation.

To fill this knowledge gap, we conducted a retrospective, multicenter study with a chemotherapy-controlled design to explore the relationship between baseline CRP levels and clinical outcomes in patients with advanced LSCC who received first-line ICI in combination with chemotherapy.

## Materials and methods

### Patients

Patients treated at 25 Chinese cancer centers between January 2016 and December 2020 were retrospectively enrolled in this study. The main inclusion criteria were: 1) a pathologically confirmed with stage IV LSCC; 2) an Eastern Cooperative Oncology Group Performance Score of 0–1; and 3) aged 18–85 years. Eligible patients received either first-line platinum-based doublet chemotherapy or ICI combined with chemotherapy. Patients with active infection, autoimmune diseases, or those taking long-term glucocorticoids were excluded from the study. Data on key clinicopathological characteristics, including sex, age, smoking status (Brinkman index), Eastern Cooperative Oncology Group performance status (ECOG PS), PD-L1 tumor proportion score (TPS, 22C3 pharmDx assay), metastasis site, CRP level at baseline, lactate dehydrogenase (LDH, considered elevated if above the individual center’s reference value), and neutrophil to lymphocyte ratio (NLR, considered elevated if ≥3) [27, 28] were collected.

### Assessments

Tumor response was assessed using the Response Evaluation Criteria for Solid Tumor (RECIST, version 1.1) [29]. The primary outcome was progression-free survival (PFS). PFS was defined as the duration from the initiation of treatment to radiographic progression or death from any cause. Patients who did not progress were recorded on the date of their last scan. The objective response rate (ORR) was defined as the percentage of patients with a confirmed complete response (CR) or partial response (PR) according to RECIST. Disease control rate (DCR) was defined as the percentage of patients with a confirmed CR, PR, or stable disease (SD) by RECIST. Overall survival (OS) was a secondary end point and OS was defined as the time from treatment to death.

### C-reactive protein

Baseline serum CRP levels were collected from the test records of patients across 25 hospitals within 1 week prior to the initiation of treatment. All centers utilized an immunological method to measure CRP. However, the reference values varied from center to center. The thresholds for considering CRP as elevated were based on these individual reference values, which are detailed in Table S1. A CRP level was deemed elevated if it exceeded the individual center’s reference value; otherwise, it was considered normal.

### Statistical analysis

Statistical analyses were performed using the R software, version 4.1.0, with R Studio software, version 1.4.1717 (R Foundation for Statistical Computing). Shapiro-Wilk normality test was applied to examine whether data samples fit a normal distribution. For the exploration of relationships among categorical clinical parameters, Chi-squared test and Fisher’s exact test were utilized, while logistic regression analysis was performed to analyze treatment efficacy. Kaplan–Meier survival curves were constructed for PFS and OS, with log-rank tests used for comparisons between patient groups. Cox proportional hazards models were employed to evaluate the effects of predictor variables on both PFS and OS. Response and its odds ratio (OR) were assessed using logistic regressions. Missing values for LDH were calculated using the chained equation method. The dose-response relationship was examined with 3-knot restricted cubic splines [30]. Prediction performance was measured by receiver operating characteristics curve (ROC) and area under ROC curve (AUC). For all statistical tests, a *p*-value below 0.05 was deemed statistically significant.

## Results

### Patient characteristics

Overall, 245 patients were included in our analysis. Among them, 140 (50.2%) received first-line platinum-based doublet chemotherapy and 105 (49.8%) received first-line chemotherapy plus ICI. Table 1 provides a summary of patient characteristics. The baseline and demographic characteristics were similar between the two treatment groups, except for modest differences in age. The median age at diagnosis was 64 years (range, 36–84 years), 224 (91%) patients were male, 229 (93%) patients were current or former smokers, and the median CRP level was 14 mg/L (interquartile range, 5–38 mg/L). Notably, a significant proportion of patients, 161 (66%), exhibited elevated baseline CRP levels.

**Table 1:** Clinical characteristics of the patient sample at the baseline

	Overall	Chemotherapy plus ICI	Chemotherapy alone	*P*
	*n* = 245	*n* = 105	*n* = 140	
**Age (years)**				
median [Range]	64 [36, 84]	65 [42, 84]	62 [36, 75]	**0.002**
**Sex (%)**				
Female	21 (9)	8 (8)	13 (9)	0.818
Male	224 (91)	97 (92)	127 (91)	
**Smoke (%)**				
Never smoker	16 (7)	8 (8)	8 (6)	0.737
Current/former smoker	229 (93)	97 (92)	132 (94)	
**ECOG score (%)**				
0	34 (14)	13 (12)	21 (15)	0.689
1	211 (86)	92 (88)	119 (85)	
**CRP (mg/L)**				
median [IQR]	14 [5, 38]	14 [3, 41]	14 [5, 37]	0.546
Normal ^a^	84 (34)	42 (40)	42 (30)	0.135
Elevated	161 (66)	63 (60)	98 (70)	
**LDH (U/L)**				
median [IQR]	196 [168, 259]	196 [172, 253]	194 [163, 273]	0.599
Normal ^a^	174 (71)	99 (71)	75 (71)	0.917
Elevated	71 (29)	41 (29)	30 (29)	
**NLR**				
median [IQR]	4 [3, 6]	4 [2, 6]	4 [3, 6]	0.285
< 3	162 (66)	72 (69)	90 (64)	0.572
≥3	83 (34)	33 (31)	50 (36)	
**PD-L1 expression on tumor cells**				
< 1%	24 (10)	17 (16)	7 (5)	0.239 ^*b*^
1–49%	32 (13)	20 (19)	12 (9)	
≥50%	15 (6)	13 (12)	2 (1)	
Unknown	174 (71)	55 (52)	119 (85)	
**Brain metastases (%)**				
Absence	223 (91)	96 (91)	127 (91)	1
Presence	22 (9)	9 (9)	13 (9)	
**Liver metastases (%)**				
Absence	199 (81)	85 (81)	114 (81)	1
Presence	46 (19)	20 (19)	26 (19)	
**Bone metastases (%)**				
Absence	168 (69)	70 (67)	98 (70)	0.677
Presence	77 (31)	35 (33)	42 (30)	
**Type of ICI**				
Pembrolizumab	38 (16)	38 (36)		
Nivolumab	25 (10)	25 (24)		
Atezolizumab	12 (5)	12 (11)		
Sintilimab	9 (4)	9 (9)		
Camrelizumab	5 (2)	5 (5)		
others	12 (5)	12 (11)		

*Abbreviations*: *CRP* C-reactive protein, *NLR* neutrophil to lymphocyte ratio, *LDH* lactate dehydrogenase, *ECOG* Eastern Cooperative Oncology Group, *PD-L1* programmed cell death 1 ligand 1, *ICI* immune checkpoint inhibitor, *SD* standard deviation; n, number of patients

^a^ Baseline CRP level and LDH level were deemed elevated if they exceeded the individual center’s reference value; otherwise, they were considered normal. ^b^ Statistics do not include the unknown

### Associations between C-reactive protein levels and progression-free survival

The median follow-up for PFS in the chemotherapy plus ICI and chemotherapy alone groups were 23.4 months (95% CI: 18.7–25.9 months) and 20.1 months (95% CI: 10.3–not estimable), respectively. In this real-world analysis, the combination treatment was superior to chemotherapy alone in improving the ORR (59% vs. 43%, OR, 0.52; 95% CI: 0.31–0.87; *p* = 0.012; Table S2) and resulted in a longer PFS (median 8.2 months vs. 5.4 months, HR, 0.48; 95% CI: 0.36–0.64; *p* < 0.001; Fig. S1).

**Fig. 1:**
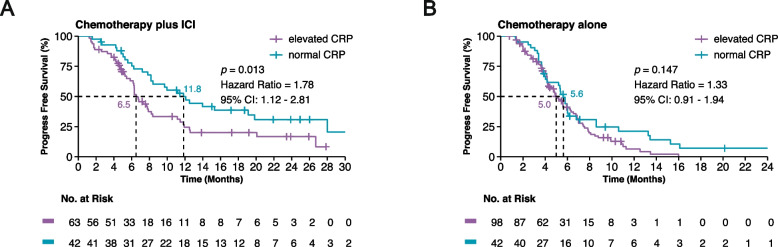
Kaplan-Meier survival curves illustrating progression-free survival (PFS). **A** Patients receiving chemotherapy plus immune checkpoint inhibitors (ICI), stratified by baseline C-reactive protein (CRP) levels (elevated vs. normal). **B** Patients receiving chemotherapy alone, stratified by baseline CRP levels (elevated vs. normal). The ‘+’ symbols represent censored data points, indicating times at which patients were lost to follow-up without experiencing the event of interest. The log-rank test was used to compare the survival distributions, and the difference was found to be statistically significant (*p* < 0.05)

In the chemotherapy plus ICI group, patients with elevated baseline CRP had a shorter PFS than those with normal CRP (median 6.5 months vs. 11.8 months, HR, 1.78; 95% CI: 1.12–2.81; *p* = 0.013) (Fig. 1A). However, CRP levels were not associated with PFS in patients treated with chemotherapy alone (median 5.0 months vs. 5.6 months, HR, 1.33; 95% CI: 0.91–1.94; *p* = 0.147) (Fig. 1B).

Following preliminary univariate analyses to evaluate the potential risk factors such as age, sex, ECOG score, LDH, NLR, PD-L1, and metastases in the brain, liver, and bone, we conducted a multivariate Cox regression analysis. This revealed that only CRP and bone metastases were significant risk factors for PFS in the combination group (Table 2). To further evaluate these two factors, we used ROC curves at 6, 12, and 18 months. The AUC values observed were 0.607, 0.661, and 0.643 for CRP, and 0.551, 0.572, and 0.544 for bone metastases, respectively (Fig. S2). These results suggest that CRP may have a better predictive accuracy.

**Table 2:** Multivariate cox regression analysis indicating significant risk factors for progression-free survival in patients treated with combination chemotherapy and immunotherapy

Characteristics	Chemotherapy plus ICI			Chemotherapy alone		
	HR	95% CI	*P*	HR	95% CI	*P*
CRP (Elevated vs. Normal)	1.84	1.13–3.00	**0.014**	1.89	1.06–3.36	**0.031**
Age (≥65 vs. < 65 years)	0.97	0.61–1.54	0.891			
Sex (Male vs. Female)	1.15	0.46–2.88	0.761			
Smoke (Current/Former vs. Never)	1.28	0.51–3.19	0.596			
ECOG score (1 vs. 0)	1.09	0.54–2.19	0.816			
LDH (Elevated vs. Normal)	1.73	1.06–2.82	**0.029**	1.44	0.80–2.58	0.225
NLR (≥3 vs. < 3)	1.19	0.72–1.96	0.500			
PD–L1 (1–49 vs. < 1%)	0.59	0.24–1.43	0.240			
PD–L1 (≥50 vs. < 1%)	0.65	0.30–1.42	0.281			
Brain metastases (Presence vs. Absence)	2.10	1.00–4.43	**0.049**	1.66	0.66–4.20	0.283
Liver metastases (Presence vs. Absence)	1.14	0.63–2.05	0.660			
Bone metastases (Presence vs. Absence)	1.74	1.08–2.8	**0.023**	1.79	1.07–3.00	**0.026**

*Abbreviations*: *CRP* C–reactive protein, *NLR* neutrophil to lymphocyte ratio, *LDH* lactate dehydrogenase, *ECOG* Eastern Cooperative Oncology Group, *HR* Hazard Ratio, *CI* Confidence Interval, *ICI* immune checkpoint inhibitor, *n*, number of patients

Bold values indicate *p* < 0.05, signifying statistical significance

For the purpose of conducting the dose-response analysis, we normalized the CRP values from each center to address variations in reference values, standardizing them to a common reference range of 0–8. In the chemotherapy plus ICI group, the restricted cubic spline analysis showed that the relationship between CRP and disease progression HR only seems to be linear at levels < 62 mg/L, with a slight decline afterwards (Fig. S3). We divided the CRP into quintiles. Patients in the fourth quintile, had a significantly poorer PFS than those in the first quintile (HR, 4.50; 95% CI: 1.85–10.95), with significant trends across quintiles (*p* = 0.039, Table S3). This suggests that while there is a clear association between higher CRP levels and poorer outcomes, the relationship is not strictly dose-dependent in a linear manner.

### Associations between C-reactive protein levels and tumor response

A summary of the efficacy results based on RECIST1.1 is provided in Table 3 In the group receiving chemotherapy plus ICI, an improved ORR was observed in patients with normal CRP compared to those with elevated CRP levels (71% vs. 51%; *p* = 0.035). Additionally, a significantly higher DCR was seen in patients with normal CRP compared to those with elevated levels (100% vs. 86%; *p* = 0.015). However, in the group receiving chemotherapy alone, no such correlations between CRP levels and either ORR or DCR were observe.

**Table 3:** Summary of treatment response in the study population: Comparison between patients treated with combination chemotherapy and immunotherapy versus chemotherapy alone

Chemotherapy plus ICI		**Overall**	**Normal CRP**	**Elevated CRP**	***P***
		*n* = 105	*n* = 42	*n* = 63	
Best Response (%)	CR	2 (2)	2 (4)	0 (0)	**0.023**
	PR	60 (57)	28 (60)	32 (51)	
	SD	34 (32)	12 (29)	22(35)	
	PD	9 (9)	0 (0)	9 (14)	
Objective Response Rate (%)	CR + PR	62 (59)	30 (71)	32 (51)	**0.035**
Disease Control Rate (%)	CR + PR + SD	96 (91)	42 (100)	54 (86)	**0.015**
**Chemotherapy alone**		**Overall**	**Normal CRP**	**Elevated CRP**	***P***
		*n* = 140	*n* = 42	*n* = 98	
Best Response (%)	CR	1 (1)	1 (2)	0 (0)	0.312
	PR	59 (42)	20 (48)	39 (40)	
	SD	66 (47)	20 (48)	46 (47)	
	PD	14 (10)	1 (2)	13 (13)	
Objective Response Rate (%)	CR + PR	60 (43)	21 (50)	39 (40)	0.322
Disease Control Rate (%)	CR + PR + SD	126 (90)	41 (98)	85 (87)	0.096

*Abbreviations*: *CR* complete response, *PR* partial response, *SD* stable disease, *PD* progressive disease, *ICI* immune checkpoint inhibitor, *n* number of patients

Bold values indicate *p* < 0.05, signifying statistical significance

Through multivariate logistic regression, CRP was discerned as a significant predictor of the ORR in patients treated with the combination of chemotherapy and immunotherapy. This relationship was not observed in the group receiving chemotherapy alone (Table S4).

### Associations between C-reactive protein levels and overall survival

**Fig. 2:**
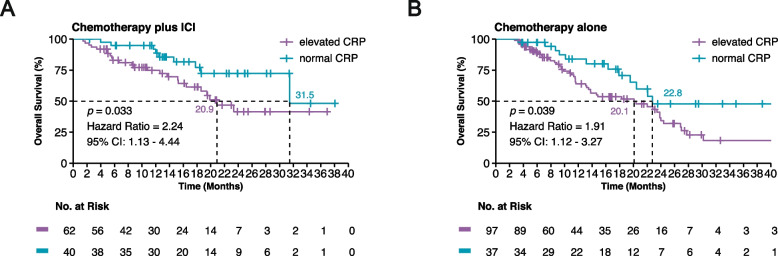
Kaplan-Meier survival curves illustrating overall survival (OS). (**A**) Patients receiving chemotherapy plus immune checkpoint inhibitors (ICI), stratified by baseline C-reactive protein (CRP) levels (elevated vs. normal). **(B)** Patients receiving chemotherapy alone, stratified by baseline CRP levels (elevated vs. normal). The ‘+’ symbols represent censored data points, indicating times at which patients were lost to follow-up without experiencing the event of interest. The log-rank test was used to compare the survival distributions, and the difference was found to be statistically significant (*p* < 0.05)

The median follow-up for OS was 18.4 months (95% CI: 16.1–22.6) and 21.2 months (95% CI: 16.9–25.9) in the chemotherapy plus ICI and chemotherapy groups, respectively. In the combination group, patients with normal baseline CRP demonstrated a substantially prolonged OS compared to those with elevated CRP (median OS 31.5 months vs. 20.9 months, HR, 2.24; 95% CI: 1.13–4.44; *p* = 0.033; Fig. 2A). In contrast, although statistically significant, the chemotherapy group presented a less pronounced difference in OS between patients with normal CRP and elevated CRP (median OS 22.8 months vs. 20.1 months, HR, 1.91; 95% CI: 1.12–3.27; *p* = 0.039; Fig. 2B).

In the multivariate Cox regression analysis, we identified CRP as an independent prognostic factor for OS in the combination chemotherapy and immunotherapy group. Conversely, CRP did not emerge as an independent factor affecting OS in the chemotherapy-alone group (Table S5).

### Relationship between C-reactive protein levels and clinical features

**Fig. 3:**
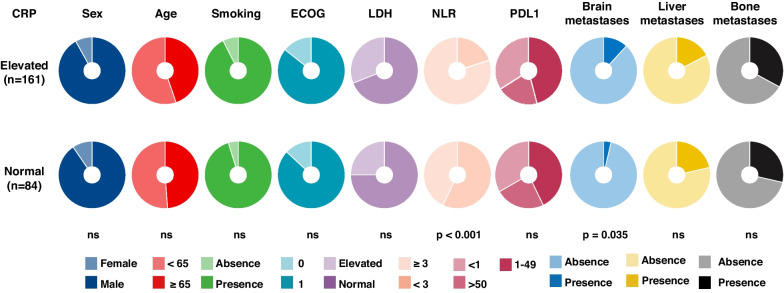
Association between C-reactive protein (CRP) and clinical characteristics. Pie charts showing the distribution of different clinicopatholotgic factors in the elevated CRP and normal CRP, respectively. The Fisher’s exact test was used to compare the difference in the proportion between the two groups. Abbreviations: Number of patients indicated (n); Not statistically significant (ns); Eastern Cooperative Oncology Group (ECOG)

In the analysis of the relationship between baseline CRP levels and clinical characteristics (Fig. 3), we identified that patients with elevated CRP levels exhibited a higher proportion of brain metastases (Fisher’s exact test, *p* = 0.035). Additionally, a significant positive correlation with the NLR was found (Fisher’s exact test, *p* < 0.001) among these patients. Contrary to expectations, considering the liver as the primary source of CRP [31, 32], no connection between elevated CRP levels and liver metastases was identified in this study.

## Discussion

To the best of our current knowledge, this study is the first to conduct a chemotherapy-controlled investigation that validates the value of CRP as a useful biomarker for advanced LSCC in patients receiving ICI in combination with chemotherapy. Our findings reveal a notable association between elevated baseline CRP levels and reduced PFS in this treatment context, a relationship not observed with chemotherapy alone, thus underscoring CRP’s specific predictive value in combination therapy. Our study also examines CRP’s role as a prognostic marker, showing its association with OS in both combination and chemotherapy-alone therapies. While this indicates CRP’s potential as a broad prognostic marker, its less pronounced impact in the chemotherapy-alone group, as highlighted by Cox model analysis, calls for a more refined understanding of its prognostic significance. These results not only provide new insights into the complex interplay between inflammatory markers and cancer treatment response, underscoring CRP’s potential as both a predictive and prognostic biomarker in LSCC, but also emphasize the necessity for further research to explore other variables influencing OS and to validate these findings in a broader range of patient cohorts.

Although several studies have shown that baseline CRP level may be a promising predictor of response to ICI treatment in advanced NSCLC [22–26], the inclusion of a control arm in our study provides further evidence to confirm the predictive nature of CRP, distinguishing it from its prognostic value. Ideally, the strongest evidence of validating predictive biomarkers would come from trials with an ‘interaction’ design. In such a study, all patients are stratified by biomarker level and then randomized to two treatments, using an interaction test to demonstrate that treatment effects differ in these two groups [33]. Given the challenges of performing randomized studies, retrospective analyses may be the most important evidence source. In the context of our findings, the predictive value of CRP appears to be especially prominent when patients are treated with chemotherapy in combination with ICI, but less so when chemotherapy is used alone. This suggests that CRP’s predictive nature might vary depending on the treatment regimen. It is noteworthy that in the chemotherapy alone group, while CRP levels did not correlate with PFS, a significant relationship with OS was observed. However, this association did not remain significant after adjusting for confounding factors. This suggests the potential influence or moderation of other variables in the relationship between CRP and OS. Further research is necessary to explore these potential relationships and interactions, offering a more comprehensive understanding of the role of CRP in cancer treatment. In addition, our study also advances the understanding of CRP as a biomarker in advanced LSCC, highlighting its complex relationship with patient outcomes in chemotherapy plus ICI therapy. We found that CRP’s predictive value for disease progression is not strictly linear, suggesting that a continuous variable analysis could be more informative than binary classification. Furthermore, given CRP’s moderate predictive performance, there is a critical need for additional or complementary biomarkers to improve clinical outcome predictions in ICI therapy.

One of the limitations of this study was that information regarding treatment-related side effects was not collected. Recent studies have suggested that high CRP predicts immunotherapy-related toxicity [34, 35]. In addition, pretreatment CRP levels may predict early death within 3 months in patients with NSCLC receiving nivolumab [36]. Based on these findings, we strongly suggest that caution should be observed regarding the use of ICI in patients with markedly high baseline CRP. The predictive value of PD-L1 expression (22C3) for pembrolizumab monotherapy response is well established [8–10]. However, histology-specific value in advanced squamous and non-squamous cancers is currently unclear. Recently, a large-scale, retrospective, real-world study of 1460 patients argued that PD-L1 may not be an appropriate predictive biomarker for checkpoint inhibitor in NSCLC with squamous histology [37]. Our study did not find a significant correlation between elevated CRP levels and PD-L1 protein expression. Interestingly, evidence from other research suggests that first-line pembrolizumab monotherapy response in patients with NSCLC and a PD-L1 TPS ≥50% tends to be poor in patients with elevated baseline CRP levels [38]. This observation highlights the potential for combined predictive utility of these markers in future research. However, the considerable amount of missing data on PD-L1 expression in our study limits the extent of this analysis. Consequently, identifying a combination of biomarkers for the highest prediction accuracy in ICI treatment efficacy remains an important area for future investigation.

Although high CRP levels have been linked to poor clinical outcomes in various types of cancers with ICI treatment, little is known about the direct effects of CRP on adaptive immunity in cancer. Recently, Yoshida et al. found that CRP inhibited the function of activated CD4+ and CD8+ T cells, induced the expression of interleukin-1β by T cells, and suppressed the expression of costimulatory molecules on mature DCs, and suppressed the expression of MART-1-specific CD8+ T cells in a dose-dependent manner, which caused an immunosuppressive environment [39]. These findings deepen our understanding of the effects of CRP on the chemotherapy plus ICI response. We also investigated other classic inflammation-related biomarkers, such as NLR or LDH, associated with ICI treatment failure in patients with NSCLC [40, 41]. However, the prognosis predictive performance of NLR and LDH was inferior to that of CRP.

Our study also has other limitations. Firstly, despite our comprehensive analysis, unaddressed unmeasured confounding factors could potentially influence the results. In particular, our dataset lacks systematic data on short-term antibiotic use, known to negatively impact PFS and OS around the initiation of ICI therapy [42]. Additionally, while we excluded patients with active infections or autoimmune diseases, the specific causes of elevated CRP levels were not identified. Moreover, our categorization of CRP levels based on each center’s criteria might not reflect the nuances of an optimal, universally applicable cutoff. However, it offers a pragmatic solution that aligns with the varying clinical practices encountered in multicentric research. Lastly, emerging evidence suggests that dynamic changes in CRP could enhance its predictive value [43, 44], implying that integrating both baseline and dynamic CRP levels, as well as considering the effects of subsequent treatments, might provide deeper insights into biomarker roles within therapeutic strategies.

## Conclusions

Our findings suggested that CRP is a useful biomarker for identifying patients unlikely to benefit from chemotherapy in combination with ICI treatment. However, further studies are needed to validate the CRP predictive value in patients with advanced LSCC, and to further elucidate the varying predictive value of CRP across different treatment modalities.

### Supplementary Information

**Additional file 1.**

## Data Availability

The data supporting this study’s findings are available on request from the corresponding author. The data are not publicly available due to privacy or ethical restrictions.
