# Supplementary material for: Baseline C-reactive protein predicts efficacy of the first-line immune checkpoint inhibitors plus chemotherapy in advanced lung squamous cell carcinoma: a retrospective, multicenter study
Source: BMC Cancer. 2023 Dec 16;23:1244. doi: 10.1186/s12885-023-11737-x (PMC10725584; doi:10.1186/s12885-023-11737-x)
Supplement: Supplementary file 1 — Additional file 1. [file 12885_2023_11737_MOESM1_ESM.pdf]

Supplementary Materials for

Baseline C-reactive protein predicts efficacy of the first-line immune checkpoint inhibitors plus chemotherapy in advanced lung squamous cell carcinoma: a retrospective, multicenter study

This PDF file includes:

Table S1 to S5
Fig. S1 to S3

Table S1. Reference Values for C-reactive protein (CRP) Across Different Hospital Centers

Center	Reference Value for CRP (mg/L)
Fujian Cancer Hospital	<10
Sir Run Run Shaw Hospital	<6
Tongji Hospital	0-8
Shengjing Hospital of China Medical University	0-6
Cancer Hospital Chinese Academy of Medical Sciences	0-6
Zhongshan Hospital Xiamen University	0-6
Fujian Provincial Hospital	0-8
The first affiliated Hospital, Zhejiang University	0-8
Peking University Cancer Hospital	<8
Fujian Medical University Union Hospital	0-8
West China Hospital, Sichuan University	<5
Sichuan Cancer Hospital	<5
Henan Cancer Hospital	0-6
Hunan Cancer Hopital	<6
Quanzhou First Hospital	0-8
Nanfang Hospital	0-6
Xijing Hospital	0-6
The Second Affiliated Hospital of Chongqing Medical University	0-10
Zhejiang Cancer Hospital	0-10
Xinqiao Hospital, Army Medical University	0-8
The Second Affiliated Hospital of Nanchang University	0-8
Jiangsu Province Hospital	0-6
The First affiliated Hospital of Xiamen University	0-10
The Second Xiangya Hospital of Central South University	0-6
Nanping First Hospital	0-10

Table S2. Tumor Response Rates in Chemotherapy Plus ICI Group versus Chemotherapy Group

	All patients (n=245)	Chemotherapy plus ICI (n=105)	Chemotherapy alone (n=140)	OR	95% CI	P
Objective Response Rate (%)	122 (50)	62 (59)	60 (43)	0.52	0.31–0.87	0.012

Abbreviations: ICI, immune checkpoint inhibitor; OR, odds ratio; CI, confidence interval; n, number of patients.

Table S3. Hazard ratios for progression-free survival according to quintile of C-reactive protein in patients

Chemotherapy plus ICI

Quintile of CRP Level	No. of Patients	Median (mg/L)	Quintile Value (mg/L)	Multivariate Analysis [a]		
				HR	95% CI	Trend *P*
1(ref)	28	1.96	< 3.98	1.00		**0.039**
2	18	6.40	3.98–10.36	1.77	0.76–4.14	
3	17	15.30	10.36–26.25	3.21	1.37–7.74	
4	18	37.79	26.25–54.74	4.50	1.85–10.95	
5	24	91.97	≥54.74	2.02	0.77–5.37	

Chemotherapy alone

Quintile of CRP Level	No. of Patients	Median (mg/L)	Quintile Value (mg/L)	Multivariate Analysis		
				HR	95% CI	Trend *P*
1(ref)	21	2.40	< 3.98	1.00		0.113
2	31	7.15	3.98–10.36	3.12	1.45–6.7	
3	32	15.60	10.36–26.25	2.36	1.25–4.43	
4	31	38.00	26.25–54.74	2.48	1.29–4.76	
5	25	84.80	≥54.74	1.53	0.79–2.95	

Abbreviations: CRP, C-reactive protein; HR, hazard ratio; CI, confidence interval; PFS, progression-free survival; ICI, immune checkpoint inhibitor.

[a] Adjusted for age, sex, smoking, PD-L1, LDH, NLR, ECOG score, brain metastases, liver metastases, and bone metastases in multivariable analysis.

Table S4. Multivariate logistic regression analysis of objective response rate in patients treated with combination chemotherapy and immunotherapy

**Characteristics**	OR	95% CI	*P*
CRP (Elevated vs. Normal)	3.27	1.23–8.71	**0.018**
Age (≥65 vs. <65 years)	1.97	0.82–4.75	0.132
Sex (Male vs. Female)	0.84	0.15–4.81	0.841
Smoke (Current/Former vs. Never)	0.85	0.16–4.54	0.853
ECOG score (1 vs. 0)	0.51	0.13–1.93	0.323
LDH (Elevated vs. Normal)	1.01	0.38–2.65	0.991
NLR (≥3 vs. <3)	0.56	0.21–1.47	0.240
PD–L1 (1–49 vs. <1%)	0.69	0.17–2.83	0.633
PD–L1 (≥50 vs. <1%)	1.45	0.31–6.70	0.612
Brain metastases (Presence vs. Absence)	0.70	0.15–3.26	0.652
Liver metastases (Presence vs. Absence)	1.59	0.52–4.91	0.427
Bone metastases (Presence vs. Absence)	0.94	0.38–2.35	0.901

Abbreviations: CRP, C-reactive protein; NLR, neutrophil to lymphocyte ratio; LDH, lactate dehydrogenase; ECOG, Eastern Cooperative Oncology Group; OR, Odds Ratio; CI, Confidence Interval; ICI, immune checkpoint inhibitor; n, number of patients.

Table S5. Multivariable analysis of factors influencing overall survival in patients treated with combination chemotherapy and immunotherapy versus chemotherapy alone

Characteristics	Chemotherapy plus ICI			Chemotherapy alone		
	HR	95% CI	*P*	HR	95% CI	*P*
CRP (Elevated vs. Normal)	2.98	1.27 – 7.00	**0.012**	1.50	0.74–3.03	0.265
Age (≥65 vs. <65 years)	1.39	0.64 - 3.03	0.406	0.85	0.29–2.50	0.762
Sex (Male vs. Female)	Inf [a]			1.49	0.84–2.65	0.173
Smoke (Current/Former vs. Never)	Inf			4.10	0.87–19.31	0.074
ECOG score (1 vs. 0)	0.82	0.28 - 2.39	0.719	1.07	0.43–2.65	0.885
LDH (Elevated vs. Normal)	2.13	0.92 - 4.89	0.075	1.13	0.65–1.97	0.665
NLR (≥3 vs. <3)	0.79	0.33 - 1.92	0.603	1.61	0.72–3.60	0.244
PD–L1 (1–49 vs. <1%)	1.10	0.27 - 4.40	0.897	1.05	0.09–12.47	0.961
PD–L1 (≥50 vs. <1%)	0.56	0.14 - 2.25	0.409	2.50	0.44–14.15	0.305
Brain metastases (Presence vs. Absence)	0.70	0.14 - 3.58	0.668	1.70	0.79–3.67	0.173
Liver metastases (Presence vs. Absence)	1.17	0.4 - 3.40	0.773	0.71	0.34–1.47	0.355
Bone metastases (Presence vs. Absence)	1.74	0.8 - 3.75	0.159	1.36	0.73–2.54	0.336

[a] The appearance of "Inf" for certain variables may indicate a lack of maturity in overall survival data or imbalances in subgroup sample sizes, leading to instability in the estimation.

Abbreviations: CRP, C–reactive protein; NLR, neutrophil to lymphocyte ratio; LDH, lactate dehydrogenase; ECOG, Eastern Cooperative Oncology Group; HR, Hazard Ratio; CI, Confidence Interval; ICI, immune checkpoint inhibitor; n, number of patients.

Fig.S1. Kaplan–Meier estimates for progression-free survival according to treatment modality

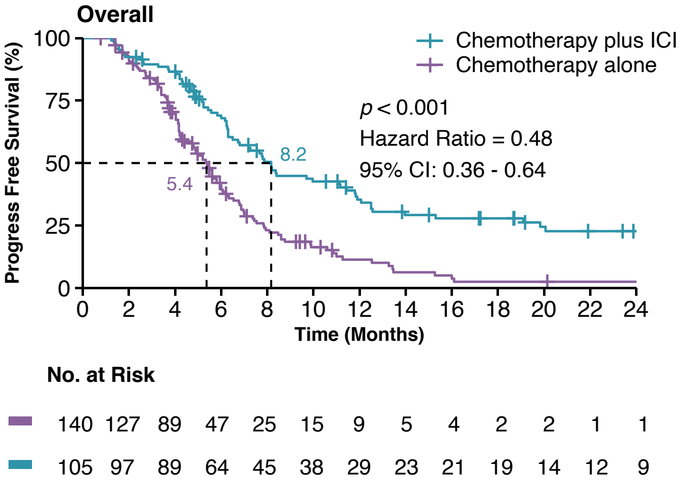

The '+' symbols represent censored data points, indicating times at which patients were lost to follow-up without experiencing the event of interest. The log-rank test was used to compare the survival distributions, and the difference was found to be statistically significant ($p < 0.05$).

Fig. S2. Comparing the Predictive Accuracy of C-Reactive Protein and Bone Metastasis for Progression-Free Survival

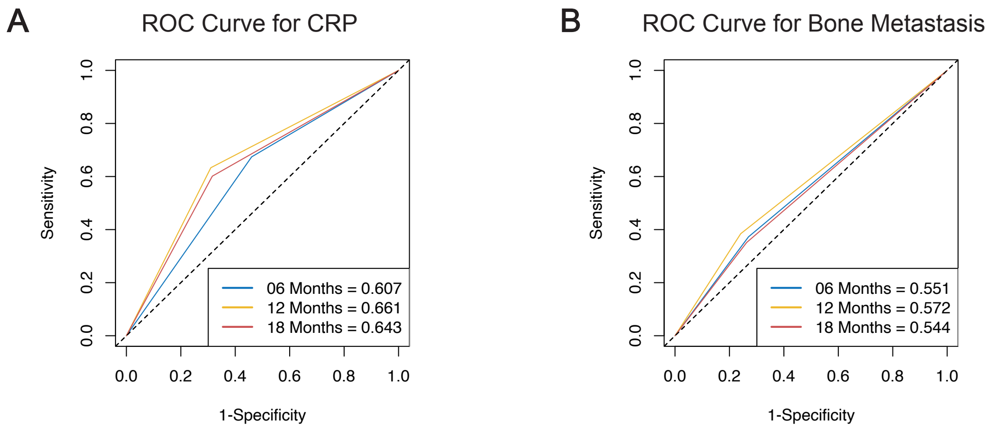

(A) and **(B)** depict the ROC curves for the prediction of Progression-Free Survival (PFS) using C-Reactive Protein (CRP) and bone metastasis, respectively. In each graph, the x-axis represents the False Positive Rate, while the y-axis represents the True Positive Rate. The Area Under the Curve (AUC), indicated next to each curve, represents the predictive performance for disease progression at 6 months, 12 months, and 18 months.

Fig. S3. Restricted Cubic Spline of the Association Between C-reactive Protein and the Disease Progression Hazard Ratio

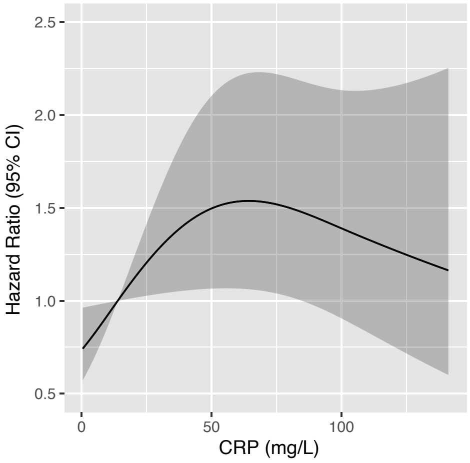

The curve is modeled using restricted cubic spline function with 3 knots.
